# A comparative study of Chinese women 3 × 3 basketball players exercise load in Tokyo Olympic preparation cycle

**DOI:** 10.3389/fphys.2023.1096423

**Published:** 2023-07-04

**Authors:** Zhe Wang, Guohuan Cao, Jiamin Xu, Jun Qiu, Ruoyu Yang

**Affiliations:** ^1^ Shanghai Research Institute of Sport Science, Shanghai Anti-Doping Agency, Shanghai, China; ^2^ Shanghai Elite Sport Training Administrative Center, Shanghai, China; ^3^ College of Rehabilitation Sciences, Shanghai University of Medicine and Health Sciences, Shanghai, China

**Keywords:** 3 × 3 basketball, exercise load, athletic performance, olympic preparation, female

## Abstract

**Objective:** The aim of this study was to compare the variances in-game loads exhibited by Chinese women’s 3 × 3 basketball team across different stages of the preparation cycle for the Tokyo Olympic Games, and to summarize the fundamental regulations governing Chinese women’s 3 × 3 basketball training and games, in order to establish a theoretical research foundation for the team’s new preparation cycle.

**Methods:** This study measured load-related data during the preparation and main competition periods of the 2019–2021 Tokyo Olympics, from April to August 2019 and from April to June 2021. The aim was to compare the changes and differences in a load of competition during different stages and to explore patterns of load changes during the preparation period. This study used wearable devices authorized by FIFA and NBA, along with the Catapult GPS performance monitoring system from Australia (Catapult&Polar Team) as instruments for collecting sports load data. The OptimEye S5 device was worn around the athlete’s neck to collect data prior to the game, while the Open Field™ system was utilized for data editing and report generation post-game.

**Results:** Compared to the primary competition load during the 2019 preparation period, the 2021 preparation period exhibited significant increases and decreases (*p* < 0.05) in competition load, high-intensity load, the number of explosive moves, the number of high-intensity acceleration, several changes to the left and right, and the number of explosive jumps. During the 2021 preparation period, the mean heart rate, mean heart rate percentage, and mean speed of the race demonstrated significant decreases in comparison to the race during the 2019 preparation period (*p* < 0.05). Throughout the training period spanning from 2019 to 2021, no significant differences were observed in running distance and maximum speed (*p* > 0.05).

**Conclusion:** The findings of this study reveal that the national training team has fostered positive adaptive changes in athletes, resulting in a significant enhancement in both load and sports performance science data during competition from 2019 to 2021.

## 1 Introduction

Basketball is a team sport that involves multiple players and has become increasingly popular among the public. Similar to the evolution of volleyball to beach volleyball and soccer to futsal soccer, a new sport has emerged based on traditional five-a-side basketball, known as three-a-side basketball (DiFiori et al., 2018; Conte et al., 2019; Ferioli et al., 2022a). Since three-player basketball is based on the traditional five-a-side basketball, it shares several techniques, tactics, and rules with the latter. However, but it also has unique features and differences that set it apart from other sports. For instance, it has a smaller court size, shorter game time, and fewer players compared to five-a-side basketball. As a result, the pace of the game is faster, and the uncertainty level is higher due to the limited number of players on the court. Three-player basketball is typically played on smaller courts, and each player has a higher workload. In contrast, five-player basketball places a greater emphasis on teamwork and tactical planning. Although there are no distinct forward or guard positions in three-player basketball each player must assume multiple roles. This results in unique sports load characteristics for three-player basketball players compared to traditional five-player basketball (García-Santos et al., 2020; Maimón et al., 2020; Ferioli et al., 2022b). These differences may have significant impacts on the physiological demands and performance characteristics of basketball. As physical load is central to athletic training, coaches and researchers can develop a variety of training contents, methods, and tools for athletes based on observed physical loads during games, within a given week.

Exercise load plays a critical role in sports training, as coaches and researchers use it to design and implement training plans for athletes. This process involves monitoring the exercise load during competition and utilizing various training methods, contents, and tools to optimize the athlete’s physical fitness and performance (Csapo et al., 2015). However, the significant differences in the sport characteristics between three-player basketball and traditional basketball have resulted in a general lack of complete understanding of the sport load of three-player basketball. This limited understanding can lead to several issues in training plans and arrangements, such as inadequate training load, insufficient intensity, and restricted ability to fully apply techniques during the game. Thus, a comprehensive understanding and accurate assessment of the athletes’ sport load characteristics during the game is crucial for effective training plans and arrangements. 3 × 3 basketball features a smaller court and fewer players, leading to higher player speeds and workloads due to increased movement distances and loads (Conte et al., 2023). Basketball studies have indicated a positive correlation between external and internal load parameters, suggesting that greater external loads lead to higher physiological and psychological demands on athletes (Willberg et al., 2022). Frequent passing and cooperation among players in 3 × 3 basketball result in more accelerations, breakthroughs, and direction changes. In contrast, 5 × 5 basketball involves a larger court, more players, and greater complexity of passing and cooperation, leading to higher heart rates and more complex movement patterns. Professional 5 × 5 basketball players have an average heart rate of 168.4 bpm and travel an average of 4.8 km, with games lasting around 40 min (Willberg et al., 2023). In comparison, 3 × 3 basketball games are shorter, lasting 10–15 min, but require higher explosive power and quick reaction ability due to the increased workload on each player (Maimón et al., 2020). The difference in sport load between the adult group and the youth group during the game is of significant importance in guiding the scientific training of high-level athletes before and after the game. This information can aid in the development of a game training plan that conforms to the developmental rules of high-level women’s basketball players, which will have a positive impact on effectively and scientifically enhancing the competitive level of Chinese three-player basketball sport. Additionally, this will contribute to the growth and advancement of Chinese three-player basketball sport.

This study focuses on the preparation of the Chinese women’s 3 × 3 basketball team for the Tokyo Olympic Games cycle. It compares the changes and differences in the game load of the team during the preparation cycle and summarizes the fundamental principles of Chinese women’s 3 × 3 basketball training and games. The study aims to provide a theoretical research foundation for the new preparation cycle of Chinese women’s 3 × 3 basketball program.

## 2 Materials and methods

### 2.1 Ethics approval

This study was conducted according to the Declaration of Helsinki and approved by the Ethics Committee of the Shanghai Research Institute of Sports Science (Shanghai Anti-Doping Center), China. Written informed consent was obtained from all participants.

### 2.2 Participants

The current study conducted research on 18 female athletes from the Chinese national 3 × 3 basketball team who underwent training from 2019 to 2021. These athletes had diverse skill levels, ranging from level-one athletes to international-level athletes. During training and competition, the athletes were segregated into two groups based on their positions on the court: interior and exterior players. Each group received different training and competition approaches. Table 1 presents comprehensive information about these athletes, such as their date of birth, height, and weight.

**TABLE 1:** Basic information about the athletes of the 2019-2021 national 3 × 3 basketball training team.

	Age/Years	Height/cm	Weight/kg
Exterior	23.9 ± 2.6	177.7 ± 3.9	69.6 ± 2.9
Interior	22.8 ± 3.1	190.1 ± 3.0	89.1 ± 3.5

### 2.3 Exercise load and performance related data collection

Test procedure: During the competition, Personalized monitoring modules (OptimEye S5, Catapult Sports, Melbourne, Australia) were worn by athletes. After the game the data were analyzed and exported using the manufacturer’s proprietary software OpenField™ system, using the PlayerLoad™ function to calculate the 3 × 3 basketball movement requirements, with the acceleration sensors collecting at 100 Hz and measuring the accumulated values of acceleration changes every 0.01 s in the X, Y, and *Z*-axes. In this study, the Polar Team system from Finland (Li et al., 2020) was employed to automatically gather data on athletes’ exercise loads. The collected data encompasses multiple indicators, such as heart rate, running distance, speed, and energy consumption. These indicators were utilized to analyze the athletes’ performance and exercise load.

Test matches were selected for this study from two main sources: 1) The 2019 National Assembled Women’s Team participated in 8 FIBA 3 × 3 Women’s Series matches and 19 matches within the 3 × 3 Basketball National Women’s team; 2) The women’s National Assembled Training Team participated in 10 competitions in Shanghai, Zhangjiagang and Chengdu stations in preparation for the Tokyo Olympic Games in 2021. All selected competitions are the main competitions in the national women’s team competitions.

### 2.4 Statistical analysis

No restrictions were imposed on any player, and each player prepared for the games based on their individual habits. Data were assessed using the IBM SPSS 26.0 software. If the data sets passed the normality test, the statistical data were expressed as mean ± standard deviation, and a two-tailed *t*-test was employed as significance test; otherwise, statistical description was based on the median and interquartile range, and a non-parametric test was performed with a significance-level of ɑ = 0.05.

## 3 Results

### 3.1 Comparative results of competition load data

Table 2 present the results of the 2021 preparation period, showing a significant increase in-game load, as well as low and high-intensity load%, whereas medium and intense load% average heart rate and average heart rate% decreased significantly. Specifically, interior players showed a significant increase in load, high-intensity load%, and maximum heart rate, while exterior players showed a significant increase in load, low and intense load%, medium intensity load%, average heart rate, and average heart rate%, with all differences statistically significant at the 0.05 level.

**TABLE 2:** Changes in national training team match load data.

	2019			2021		
	interior	exterior	all	interior	exterior	all
Load capacity Player Load™/au	118.83 ± 24.45	136.67 ± 31.14	129.73 ± 29.91	147.78 ± 15.84**	153.50 ± 27.87*	150.93 ± 23.15**
Player Load™.min/au	6.36 ± 1.29	7.53 ± 1.28	7.08 ± 1.40	6.76 ± 0.59	7.24 ± 1.04	7.02 ± 0.89
Low-intensity loads (%)	76.07% ± 3.81%	74.35% ± 4.87%	75.02% ± 4.55%	76.17% ± 2.62%	77.27% ± 3.69%**	76.78% ± 3.26%*
Medium-intensity loads (%)	22.00% ± 3.71%	21.89% ± 4.35%	21.94% ± 4.09%	20.83% ± 3.01%	17.96% ± 3.47%**	19.25% ± 3.54%**
High-intensity loads (%)	1.83% ± 0.82%	3.67% ± 1.71%	2.95% ± 1.69%	2.72% ± 1.23%*	4.73% ± 1.67%*	3.83% ± 1.78%**
Average heart rate (bpm)	158.61 ± 12.41	168.68 ± 8.16	164.09 ± 11.43	154.30 ± 10.08	159.14 ± 9.14*	157.08 ± 9.96*
Maximum heart rate (bpm)	181.98 ± 9.69	195.02 ± 13.09	189.07 ± 13.31	187.78 ± 19.15	190.64 ± 14.77	189.43 ± 16.73
Average heart rate (%)	80.12 ± 6.41	85.5 ± 4.40	83.04 ± 6.01	79.22 ± 5.23	80.30 ± 4.47*	79.84 ± 4.92*
Maximum heart rate (%)	91.76 ± 5.29	98.82 ± 6.94	95.60 ± 7.14	79.22 ± 5.23*	96.24 ± 7.67	96.31 ± 8.47

Note:* represents a significant difference of *p* < 0.05 compared to 2019; data from Catapult and polar systems.

### 3.2 Match performance data comparison results

Table 3 illustrate that the team demonstrated a noteworthy improvement in various physical performance indicators in 2021, such as explosive movement counts, high-intensity acceleration, and counts in the leftward and rightward directions. Furthermore, the team displayed a significant reduction in their average speed and explosive jumping. Meanwhile, the interior exhibited an increase in explosive movement counts and high-intensity acceleration while experiencing a significant decrease in explosive jumping. The exterior, on the other hand, demonstrated a significant increase in high-intensity acceleration, counts in the leftward and rightward directions, and average speed, along with a significant decrease in explosive jumping. All of these results showed statistically significant differences at the 0.05 level.

**TABLE 3:** Comparative results of performance figures for the whole team.

	2019			2021		
	interior	exterior	all	interior	exterior	all
Distance in speed zone 4 [m]	40.29 ± 25.50	76.82 ± 41.49	60.14 ± 39.43	36.89 ± 33.85	73.70 ± 38.90	58.05 ± 40.95
Distance in speed zone 5 [m]	6.71 ± 7.65	45.32 ± 155.76	27.70 ± 116.03	10.13 ± 15.24	33.48 ± 32.77	23.55 ± 29.05
Total distance [m]	956.19 ± 224.25	1060.60 ± 299.59	1012.94 ± 271.51	964.41 ± 246.87	987.86 ± 251.95	977.89 ± 248.63
Max. speed [km/h]	20.41 ± 2.02	22.66 ± 2.30	21.63 ± 2.44	20.03 ± 3.15	23.41 ± 2.32	21.98 ± 3.17
Average speed [km/h]	3.51 ± 0.47	3.75 ± 0.61	3.64 ± 0.56	3.28 ± 0.46	3.47 ± 0.61*	3.39 ± 0.56*
Number of bursts of movement (>3.5 m.s)	19.76 ± 9.27	26.14 ± 9.86	23.66 ± 10.09	26.11 ± 6.43**	36.14 ± 10.84*	31.63 ± 10.33**
High-intensity acceleration (>3.5 m.s)	1.95 ± 3.21	6.52 ± 3.58	4.74 ± 4.09	8.00 ± 3.40**	11.14 ± 3.48**	9.73 ± 3.75**
High-intensity deceleration (>3.5 m.s)	4.21 ± 2.59	5.41 ± 3.17	4.94 ± 3.00	4.22 ± 2.94	5.27 ± 2.99	4.80 ± 2.98
High-intensity to the left (>3.5 m.s)	4.76 ± 4.26	7.18 ± 3.89	6.24 ± 4.19	6.83 ± 2.96	9.14 ± 3.97*	8.10 ± 3.69*
High-intensity to the right (>3.5 m.s)	6.17 ± 3.07	7.08 ± 3.15	6.72 ± 3.14	7.06 ± 2.58	10.59 ± 4.03**	9.00 ± 3.85**
Explosive jumps (>40 cm)	3.36 ± 2.86	2.38 ± 2.76	2.76 ± 2.83	0.78 ± 1.56**	1.55 ± 1.60	1.20 ± 1.60**

Note:* represents a significant difference of *p* < 0.05 compared to 2019; number of explosive moves = high intensity acceleration + high intensity deceleration + high intensity to the left + high intensity to the right; data from Catapult and polar systems.

## 4 Discussion

This study employed GPS wearable monitoring systems to collect and analyze data from Chinese women’s 3 × 3 basketball games during the period of 2019–2021. The objective was to analyze and summarize the game load and performance characteristics of the players. Specifically, the study aimed to investigate the changes in-game load during different stages of the preparatory period and identify factors that may affect these changes. The overarching goal was to optimize load control and improve the athletes’ performance level and game results. The study aimed to provide theoretical and practical guidance for training plans and load control during the preparatory period, with the ultimate goal of enhancing the competitive level of Chinese women’s 3 × 3 basketball. Therefore, the study sought to reveal load changes and performance characteristics of female 3 × 3 basketball players in games, with the aim of supporting the improvement of their competitive level.

The average and maximum heart rates observed during 3 × 3 basketball games indicate that this sport involves a moderate to high level of intensity. An examination of the physiological demands placed on the athletes revealed that the repetition of intermittent sprints during exercise necessitates the utilization of both anaerobic glycolysis and aerobic pathways to provide energy. For female athletes, the average heart rate during these games was recorded as 164 ± 12 beats/min (Montgomery and Maloney, 2018a). The findings of this study align with the data collected from April to August 2019, with a subsequent decrease in performance observed at 21 years of age. This observation may be attributed to the effective adaptation of national training team athletes to the rigorous demands of high-intensity 3 × 3 basketball games, along with the strategic transition and substitution rotation tactics employed by the team to optimize internal loads while enhancing athletic performance. It is worth noting, however, that the competition line-ups have undergone modification since 2019, with the national red and blue team line-ups shifting from a composition of 1 interior and 3 exterior players to 2 interior and 2 exterior players, thereby increasing the height of the team line-ups.

To sustain fatigue resistance among athletes engaged in 3 × 3 basketball, a targeted approach toward enhancing both aerobic and anaerobic capacity is required (Montgomery and Maloney, 2018a). In contrast to five-a-side basketball, where players possess innate physical and physiological traits, such as height, body mass, arm span, and muscle mass, that enable them to fulfill specific roles as guards, forwards, and centers (Hoffmann et al., 2014), the three-player basketball program blurs positional distinctions. Nonetheless, aerobic capacity remains an essential physiological characteristic for high-performing basketball players.

The investigation ascertained that 3 × 3 basketball places heightened emphasis on explosive and swift techniques and movements, relative to conventional basketball. Consequently, it is suggested that 3 × 3 basketball training ought to concentrate on elevating high-intensity anaerobic capacity, which encompasses rapid acceleration and deceleration, explosive endeavors, jumping, and directional changes, while simultaneously prioritizing the enhancement of aerobic capacity, to bolster the athletes’ ability to sustain optimal performance throughout the game. This may be accomplished through high-intensity interval training, involving the alternation of high-intensity aerobic exercises, such as rapid running or cycling, and high-intensity anaerobic exercise, such as swift explosive movements. Furthermore, given that the customary offensive duration in 3 × 3 basketball is exceedingly brief, it is essential that athletes possess rapid recovery capability, to complete multiple high-intensity movements within a truncated timeframe. As a result, comprehensive training of aerobic and anaerobic capacity, along with swift recovery ability, is critical to elevate the performance of 3 × 3 basketball athletes (Willberg et al., 2023).

The enhancement of the national team’s performance during the 2019–2021 biennium could be attributed to various factors, such as an improved level of training, successful adaptation to the training and competition routines of the three-player basketball program, and the incorporation of targeted training tools, including general endurance training and load monitoring equipment to track variables such as heart rate during daily training sessions. Such measures have contributed to lower heart rate loads and may have influenced the team’s ability to adapt to new demands. Studies have indicated that the Yo-Yo test is a valid tool for evaluating the aerobic capacity of basketball players. This is because skeletal muscle tissue requires a substantial amount of oxygen during physical activity, and the maximal oxygen uptake reflects the body’s capacity to uptake oxygen. Recent research suggests that high-intensity interval training (HIIT) can benefit young athletes by improving both aerobic and anaerobic performance, while also reducing the amount of time required for specific training (Difiori et al., 2018; Engel et al., 2018). To optimize training quality, it may be worthwhile to incorporate more HIIT training programs specifically designed for enhancing aerobic and anaerobic capacity in the future.

Compared to 2019, there was a significant increase observed in the load and the percentage of high-intensity load in the 2021 competitions. This observation may be attributed to the enhanced overall strength of the national training team, as a result of an extended period of training and competition, leading to an increase in the intensity of the competition load. Previous studies on elite male and female 3 × 3 basketball players reported athlete loads of 127.5 ± 31.1 au and 128.5 ± 32.0 au, respectively (Montgomery and Maloney, 2018a; Montgomery and Maloney, 2018b). The elevated athlete load observed in this study compared to the aforementioned earlier studies implies that the competition level and athlete performance level of the 2019-2021 national team is even more advanced than previously assessed. As the level of competition in 3 × 3 basketball increases, so does the intensity and load of the game. The national team prioritizes adapting and enhancing the specific characteristics of the three-player basketball event during regular training. Emphasis is placed on elevating the intensity of team confrontation via 1v1, 2v2, and 3v3 scenarios. The data for the 2021 team primarily stems from the training tournament, which implemented a format of multiple matches per day to simulate the Tokyo Olympics. During this tournament, the national team was split into two teams, designated as the red and blue teams, to facilitate training and problem identification through repeated matches. This approach further enhanced the overall quality of training. Load monitoring is commonly conducted in team programs to measure the physical demands placed on basketball players, and various devices such as heart rate monitors, rating of perceived exertion (RPE) scales, real-time motion analysis, local positioning systems, and accelerometers are utilized for this purpose (Reina et al., 2020; Sanders et al., 2021; Power et al., 2022). In light of this, in order to enhance training methodologies and tools, it is imperative to undertake load testing for 3 × 3 basketball in the future.

Between 2019 and 2021, there was a substantial rise in the frequency of explosive movements and directional changes during gameplay. This trend may be attributed to a marked elevation in the frequency of explosive movements and directional transitions in all dimensions, alongside a notable escalation in high-intensity load proportion and athletes’ exposure to more intense confrontations. Specifically, this phenomenon could be associated with the daily training regimen of the national training team, which aims to enhance athletic performance through increasing movement frequency, targeted training in agility strides, and tailoring individualized weight control programs for each athlete to reduce body fat percentage and augment muscle mass.

With regard to jumping proficiency, existing literature suggests that the typical vertical jump height for female basketball players ranges from 22 to 48 cm (Ziv and Lidor, 2010), and superior basketball players generally exhibit higher vertical jump height than their non-basketball athlete counterparts (Hoare, 2000). The findings of this investigation indicated that, during the 2021 basketball season, there was a marked decrease in the number of explosive jumps exceeding 40 cm among players as compared to non-players. This may be attributed to the shift in the starting lineup, with a move from a formation of one interior and three exterior players to a dominant form of two interior and two exterior players. This alteration likely presented physical challenges in executing more forceful jump-based offensive and defensive maneuvers while adapting to the high-intensity nature of the game. Repetitive high-speed movements, such as accelerating, decelerating, turning, and jumping, require a confluence of strength, speed, and agility training, along with technical proficiency in shooting and passing, in order to attain peak performance outcomes (Montgomery and Maloney, 2018b). Assessments of elite basketball players have demonstrated that guards and forwards tend to possess superior aerobic and relative anaerobic capacity, resulting in shorter recovery times and enhanced ability to perform repeated high-intensity movements. Conversely, centers generally exhibit greater absolute anaerobic power and explosive power, facilitating heightened power generation in discrete activities, such as jumping (Taylor et al., 2017). Therefore, this implies that basketball coaches and physical trainers should augment their training programs to incorporate greater specificity in relation to jumping ability in the future.

The present investigation did not identify any notable alteration in the distance covered during games played between 2019 and 2021, whereas a noteworthy escalation in the average speed was observed. This observation also indicates that women’s 3 × 3 basketball players are enhancing their performance, as they concentrate more on developing their individual skills and displaying greater involvement in the game, which leads to the creation of opportunities through sustained running. There are several possible reasons for this transformation, including the implementation of personalized training plans tailored to individual requirements, the optimization of training intensity and workload for both the team and individual players, enhanced player recovery programs, routine screening of players for underlying physical issues and deficiencies via functional assessments, emphasis on stretching, activation, and relaxation techniques, and improved interval hydration and post-training nutritional practices. These factors may have played significant roles in the national 3 × 3 basketball women’s team winning the bronze medal at the Tokyo Olympics. Presently, elucidating the physical and physiological requirements of high-level 3 × 3 basketball can assist coaches and athletes in optimizing game and training loads through targeted training. However, it is imperative to combine relevant scientific research with the foundation of previous studies to conduct long-term and longitudinal comparative analysis.

After conducting research, it has been determined that monitoring athlete workload using GPS sensors has demonstrated advantages in terms of effectiveness compared to standard instruments based on motion capture systems (Jimenez-Olmedo et al., 2023). Currently, heart rate indicators are widely used for monitoring internal training load; however, future research may consider incorporating the Rating of Perceived Exertion (RPE) scale as a measurement tool. It should be noted that RPE and heart rate indicators are not linearly related, as evidenced by previous studies (Lupo et al., 2017). Additionally, relevant research has demonstrated that heart rate and time-motion parameters used in other team sports such as soccer, track and field, cycling, and running have practical value. These parameters can be utilized to measure athletes’ movement trajectories, distance, speed, acceleration, and other indicators of physical load, which in turn can aid in training and competition and assist athletes in developing relevant tactics (Lupo et al., 2016). Therefore, in future research, appropriate monitoring indicators and devices should be selected to provide more precise data support for athletes’ training and competition.

In conclusion, between 2019 and 2021, the Chinese national training team has made significant progress in terms of game load and scientific data on athletic performance, with athletes demonstrating favorable adaptive changes to game load. The data from this period reveal a noteworthy enhancement in the level of play of Chinese women’s 3 × 3 basketball, as evidenced by improvements in-game load, technical movements, and confrontational ability. Consequently, future research should not only continue to observe further changes in Chinese 3 × 3 basketball players’ adaptations to the sport, but also devise more effective training strategies based on their current adaptations to the sport in order to improve their performance.

## Data Availability

The raw data supporting the conclusion of this article will be made available by the authors, without undue reservation.
